# The effects of acupuncture on cognitive deficits in transgenic mouse studies of mild cognitive impairment and Alzheimer's disease: Study protocol of a systematic review

**DOI:** 10.1097/MD.0000000000017557

**Published:** 2019-10-18

**Authors:** Yang Yang, Shaowen Hu, Jiang He, Jianguo Zhang, Chunzhi Tang

**Affiliations:** a Clinical Medical College of Acupuncture and Rehabilitation, Guangzhou University of Chinese Medicine, Guangzhou; b Ruikang Hospital Affiliated to Guangxi University of Chinese Medicine, Nanning, China.

**Keywords:** acupuncture, Alzheimer's disease, mild cognitive impairment, systematic review, transgenic mouse

## Abstract

**Background:**

Mild cognitive impairment (MCI) and Alzheimer's disease (AD) are neurodegenerative diseases associated with aging. The major clinical features of both are progressive memory loss and progressive cognitive loss. The objective of this systematic review protocol is to provide the methods for evaluating the effectiveness of acupuncture in the treatment on cognitive deficits in transgenic mouse.

**Methods and analysis:**

We will search the electronic databases of PubMed, Web of Science, Embase, PsycINFO, as well as the Chinese databases such as Chinese Biomedicine Literature (CBM), Chinese Medical Current Content (CMCC), Chinese Scientific Journal Database (VIP), WanFang Database and China National Knowledge Infrastructure (CNKI) from their inceptions to July 2019. RevMan 5.3 software will be used for the data synthesis and the quality of each study was assessed independently by use of the CAMARADES checklist.

**Results:**

This review will provide a high-quality synthesis based on present evidence of acupuncture treatment for AD and MCI in transgenic mouse models.

**Conclusions:**

This systematic review will provide evidence for weather acupuncture is an effective intervention for AD and MCI in transgenic mouse models.

**Ethics and dissemination:**

Ethical approval is not necessary since this protocol is only for systematic review and does not involve privacy data or conduct an animal experiment. This protocol will be disseminated by a peer-review journal or conference presentation.

**Trial registration number:**

PROSPERO CRD42019142985.

**Strengths and limitations of this study:**

This systematic review will be the first to provide new knowledge underlying the effectiveness to improve cognitive function of acupuncture treatment for AD and MCI in transgenic mouse models. The result of this systematic review may provide experimental and theoretical basis for the future clinical application of acupuncture in the treatment of AD.

The limitation of this systematic review may come from language barriers, because only English and Chinese can be included. Also, this study includes various kinds of acupuncture treatments which may result in essential heterogeneity.

## 1 Introduction

Alzheimer's disease (AD) is an age-related neurodegenerative disease characterized by progressive memory and neuronal loss combined with cognitive impairment.^[1]^ It is the most common neurodegenerative disease in the world.^[2]^ Mild cognitive impairment (MCI) is considered to be a prodromal phase (preclinical stage) of AD.^[3]^ The early definition of MCI refers to the progressive memory impairment and cognitive impairment similar to AD, However, it retains the overall cognitive function and activities of daily living.^[4,5]^ Mild cognitive impairment and AD are neurodegenerative diseases associated with aging. The major clinical features of both are progressive memory loss and progressive cognitive loss.^[6,7]^ However, the pathogenesis has not been well elucidated, which limits its pharmacological treatment.^[8]^ MCI patients are at high risk of developing AD. Studies have shown that the annual conversion rate of MCI to AD is about 8.3% to 28%,^[5,9]^ and the rate of conversion to AD increases with age and course of disease, while the rate of normal elderly people is only 1% to 2%.^[10]^

With the rapid development of the global economy and the improvement of the public basic health conditions, the average life span of human beings is increasing, which will undoubtedly lead to the aging of the world population. It is expected that there will be global MCI, AD and AD-related dementia pandemics.^[11,12]^ According to the data released by Alzheimer's Disease International in 2018, as of 2015, there were about 50 million AD patients in the world, and the number will reach about 152 million in 2050. The total cost of treatment for Alzheimer's patients worldwide is as high as $1 trillion in 2018, and by 2030, this number will double.^[2]^

So far, the clinical treatment of AD is mainly western medicine according to the clinical guidelines. The US Food and Drug Administration has approved five drugs, including donepezil, rivastigmine, galantamine, and tacrine (four acetyl cholinesterase inhibitors) and memantine (an N-methyl-D-aspartate receptor antagonist) for the treatment of AD.^[13,14]^ However, these drugs can only alleviate the symptoms of learning, memory, and cognitive impairment. No drugs have been found to completely cure AD or improve the life quality of patients. Moreover, chemical drugs have many shortcomings certain adverse reactions, such as large side effects, high price, poor compliance, and unclear efficacy. Due to the limitations of the existing drugs, the need for clinical intervention in patients with AD and MCI is particularly important and urgent, so it is of great significance to find a breakthrough non-pharmacological therapy.^[15,16]^

As a non-pharmacological treatment method of traditional Chinese medicine, acupuncture has been applied to the treatment of dementia since ancient times. Moreover, acupuncture has advantages such as safety, convenience, very few side effects, and low price. A large number of clinical studies suggest that acupuncture can significantly improve cognitive function and improve patients’ quality of life.^[17–19]^ In recent years, more and more animal researches have been made to explore the mechanism of acupuncture treatment of AD and a large number of studies have confirmed that acupuncture can improve the cognitive function of AD model animals, providing experimental and theoretical basis for the future clinical application of acupuncture in the treatment of AD and MCI.^[20–23]^ However, there is still remaining no systematic review to present evidence of effectiveness of acupuncture for reducing cognitive dysfunction in AD transgenic mouse models. Consequently, our study is performed to provide a systematic review in terms of the efficacy of acupuncture in the treatment of AD and MCI in transgenic mouse.

## 2 Methods

### 2.1 Study registration

This systematic review protocol has been registered on PROSPTERO (www.crd.york.ac.uk/prospero/) with number CRD42019142985. The protocol follows a checklist of the Collaborative Approach to Meta-Analysis and Review of Animal Data from Experimental Studies (CAMARADES) with minor modifications.^[22]^ We will describe the changes in our full review.

### 2.2 Inclusion criteria for study selection

#### 2.2.1 Types of studies

Only the controlled studies with a separate control group will be included. Languages including English and Chinese, and Chinese will be translated into English in the final version. No publication dates limitations.

#### 2.2.2 Types of participants

All transgenic mouse models with MCI or AD (all sexes).

#### 2.2.3 Types of interventions

All transgenic mouse models with MCI or AD which used acupuncture and related techniques alone as intervention, such as acupuncture, electroacupuncture, moxibustion, acupressure, cupping, acupoint injection, laser acupuncture, auricular needle, scalp needle, acupoint bloodletting therapy, fire needling, intradermal needling and acupoint catgut embedding acupressure, etc. All timings, frequencies and duration of treatment are eligible for inclusion.

#### 2.2.4 Types of outcome measures

Morris water maze, passive avoidance test, Y-maze test and other behavior test which is commonly used to evaluate spatial learning and memory ability in mouse models.

### 2.3 Exclusion criteria

1. Animals other than transgenic mouse.
2. Vivo, in vitro, and in silico models.
3. Case studies, cross-over studies, studies without a separate control group.
4. Cognitive dysfunction in mice is derived from other diseases, not MCI or AD.
5. The intervention was acupuncture combined with Chinese herbal medicine or western medicine or any other type of treatment.
6. The intervention was aimed to compare different acupuncture techniques or different acupoints.
7. No relevant outcomes reported.
8. Duplicate publications.

### 2.4 Search methods for identification of studies

#### 2.4.1 Electronic searches

We will search the following electronic databases from inception to July 2019: PubMed, Web of Science, Embase, PsycINFO, as well as the Chinese databases such as Chinese Biomedicine Literature (CBM), Chinese Medical Current Content (CMCC), Chinese Scientific Journal Database (VIP), WanFang Database, and China National Knowledge Infrastructure (CNKI). Databases were searched from their inception dates to July 2019. No language restrictions were applied in the search strategy.

#### 2.4.2 Searching strategy

The search strategy for Pubmed is listed in Table 1, which includes all search terms, and other searches will be conducted based on these results. This search strategy will be modified as required for other electronic databases.

**Table 1:**
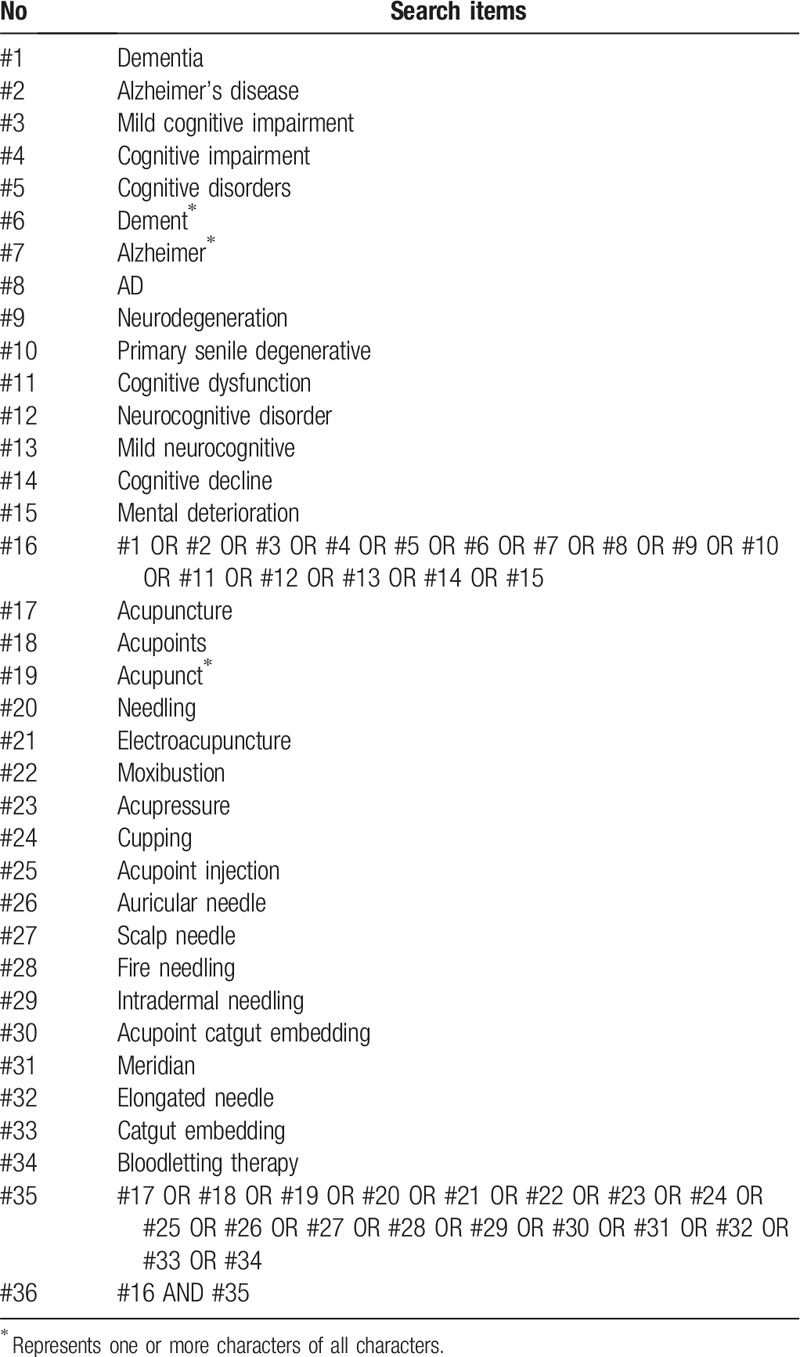
PubMed search strategy.

### 2.5 Data collection and analysis

#### 2.5.1 Selection of studies

**Figure 1:**
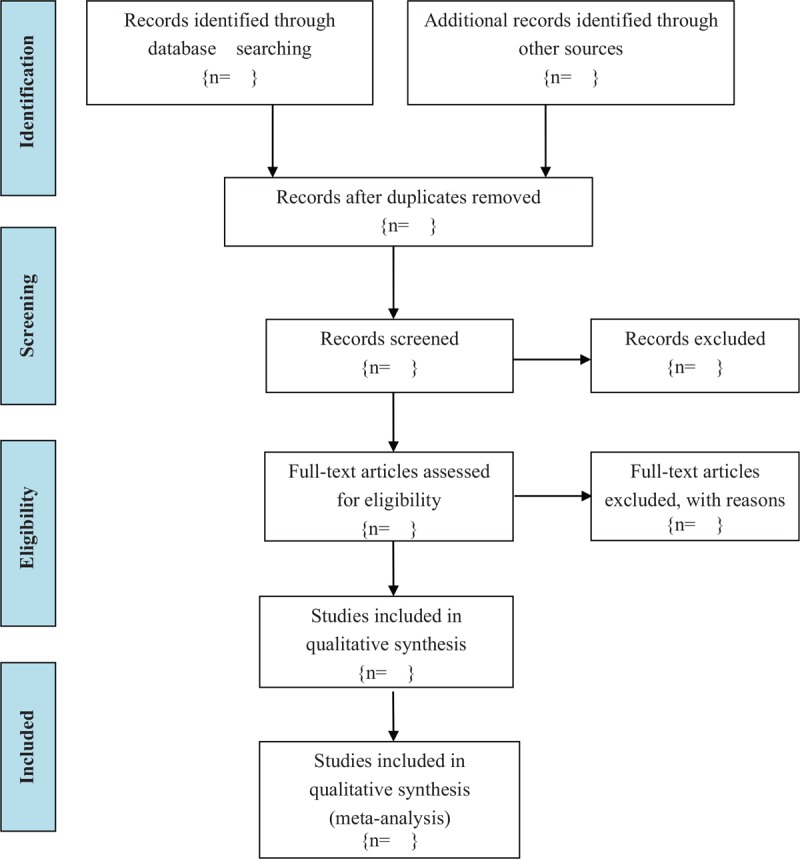
Flowchart of study selection.

To select studies for this review, two researchers (Yang Yang and Shaowen Hu) evaluate the titles and abstracts of identified articles. Then, the two researchers download the full text of the articles to review the study design and methodology for studies that used acupuncture and its related techniques as intervention and measure cognitive problems in transgenic mouse. Each researcher will make decision on every research independently. Disagreements among the researchers will be resolved by a discussion. If they do not reach the same decision, the concerned articles will be discussed with a third author (Chunzhi Tang) until the consensus is reached. The whole procedure of study selection will be performed according to the Preferred Reporting Items for Systematic Reviews and Meta-Analysis (PRISMA) flow chart in Figure 1.

#### 2.5.2 Data extraction and management

Two researchers (Yang Yang and Shaowen Hu) will be responsible for data extraction. They must agree on the eligibility criteria. If the data is presented graphically, the researchers will attempt to obtain the numerical values from the trial authors; if the value is not available, we will use the digital ruler software to estimate the values in the graph. If we are unable to obtain complete data, then we will exclude the test of missing data from the data synthesis. If there are any disagreements, the authors will discuss with each other and ask the third author (T.CZ.) to resolve the differences until a consensus is reached.

#### 2.5.3 Assessment of risk of bias and reporting of study quality

The quality of each study was assessed independently by use of the CAMARADES checklist, they will get a score from 0 to 10. Two reviewers (Yang Yang, Shaowen Hu) independently extracted the data and assessed the quality of each study. Disagreements were resolved through discussion.

#### 2.5.4 Measurement of treatment effect

We will use the relative risk with 95% confidence intervals (CIs) to analyze the effects of treatment for dichotomous outcomes. For continuous outcomes, we will use the weighted mean difference or the standard mean difference (SMD) with 95% CIs. The SMD is equal to the difference in the mean outcome between the groups divided by the standard deviation of the outcomes among the participants, which is reported in units of standard deviation and allows data measured on different scales to be merged. Negative SMD effect sizes indicate a positive efficacy for memory acquisition, the other is the opposite.

#### 2.5.5 Unit of analysis

For studies comparing different type, timing or duration of acupuncture treatment to a single control group, the data from all experimental groups will be pooled to compare with the control group. For crossover trials, we will extract the data of first study period to avoid the unit of analysis issue.

#### 2.5.6 Dealing with missing data

If the extractions are unclear or incomplete, we will try to contact the corresponding author or first author to obtain the data. If the data cannot be obtained, the article will be excluded.

#### 2.5.7 Assessment of heterogeneity

Both the *χ*^2^ test and the *I*^2^ statistic will be used for the assessment of heterogeneity between the studies in effect measures. We will consider an *I*^2^ value > 50% suggests unacceptable heterogeneity.

#### 2.5.8 Assessment of reporting bias

If there are more than 10 studies included in this meta-analysis, the funnel plot is drawn to detect if there is a publication bias; if <10 studies are included, there is no need to draw a funnel plot, and it is not necessary to check the asymmetry of the funnel plot.

#### 2.5.9 Data synthesis

The meta-analysis will be performed with RevMan 5.3 software. *P* values of < .05 were considered statistically significant. We will select the fixed-effects model (*I*^2^ < 50%) or random-effects model (*I*^2^≥50%) based on the heterogeneity levels of the included studies. Sensitivity analysis and subgroup analysis will also be performed to find out any possible reasons that may cause the heterogeneity. However, we will not accept the data in meta-analysis if significant heterogeneity (*I*^2^ > 75%) still exists after sensitivity analysis and subgroup analysis.

#### 2.5.10 Subgroup analysis

Subgroup analyses will also be used to identify associations between relevant study characteristics, such as different type of transgenic mouse, sex, anesthetic method, type and duration of acupuncture, and study quality, when substantial heterogeneity existed.

#### 2.5.11 Sensitivity analysis

After conducting a quality assessment of the included studies, we will conduct a sensitivity analysis if there are studies of low quality. Sensitivity analysis will also be performed when heterogeneity testing suggests significant heterogeneity between studies.

## 3 Discussion

AD is an age-related neurodegenerative disease. The prevalence of AD doubles every 5 years in people aged 65 to 85, from about 1% to 2% at age 65 to more than 30% to 50% at age 85.^[24]^ And these are only the cases that are clearly diagnosed by doctors. In developed countries, only 50% of AD patients are definitely diagnosed, and in developing countries, even <10%.^[4]^

Clinically, cognitive impairment, learning and memory dysfunction, and mental disorders are the main clinical manifestations and diagnostic indicators in patients with AD and MCI. Therefore, animal experiments are often conducted to test whether the behavioral changes of animal models with cognitive dysfunction are consistent with clinical symptoms in human, so as to evaluate the reliability of animal models and the effectiveness of therapeutic intervention methods. In recent years, experimental studies on cognitive impairment using transgenic mouse have been increasing. Also, and transgenic mouse has been recognized as the animal model closest to AD.^[25,26]^

Acupuncture is frequently used in China and believed to affect memory and learning in AD and MCI. There are numerous clinical studies demonstrate that acupuncture has positive effect in improving the cognitive function of MCI and AD. But the underlying mechanism of acupuncture in treating cognitive impairment has not yet been found. So there is more and more animal studies conducted to explore the underlying mechanism. Currently, no systematic review and meta-analysis have been conducted regarding the effectiveness to improve cognitive function of acupuncture treatment for AD and MCI in transgenic mouse models. Then we perform a meta-analysis of published AD transgenic mouse studies which used acupuncture as intervention to improve cognitive performance to examine the potential correlation between acupuncture and cognitive function caused by MCI or AD.

## Acknowledgments

This work was supported by the National Natural Science Foundation of China (No.8187151425).

## Author contributions

**Conceptualization:** Chunzhi Tang, Shaowen Hu.

**Data curation:** Shaowen Hu, Jiang He, Jianguo Zhang.

**Investigation:** Yang Yang.

Jiang He, Jianguo Zhang.

**Methodology:** Yang Yang, Shaowen Hu.

**Project administration:** Chunzhi Tang, Yang Yang.

**Resources:** Chunzhi Tang, Shaowen Hu.

**Supervision:** Yang Yang.

**Writing – original draft:** Yang Yang, Shaowen Hu,

**Writing – review & editing:** Chunzhi Tang, Yang Yang.

## References

[1] Goedert M Spillantini MG A century of Alzheimer's disease. Science (New York, NY) 2006;314:777 81. 10.1126/science.1132814 17082447

[2] Patterson C World Alzheimer Report 2018. The State of the Art of Dementia Research: New Frontiers. 2018;London: Alzheimer's Dis Int, 1–48.

[3] Petersen RC Smith GE Waring SC Mild cognitive impairment: clinical characterization and outcome. Arch Neurol 1999;56:303 8. 10190820 10.1001/archneur.56.3.303

[4] Petersen RC Doody R Kurz A Current concepts in mild cognitive impairment. Arch Neurol 2001;58:1985 92. 11735772 10.1001/archneur.58.12.1985

[5] Petersen RC Morris JC Mild cognitive impairment as a clinical entity and treatment target. Arch Neurol 2005;62:1160 3. 16009779 10.1001/archneur.62.7.1160

[6] Huang Y Mucke L Alzheimer mechanisms and therapeutic strategies. Cell 2012;148:1204 22. 22424230 10.1016/j.cell.2012.02.040 PMC3319071

[7] Shin JY Park HJ Kim HN Mesenchymal stem cells enhance autophagy and increase beta-amyloid clearance in Alzheimer disease models. Autophagy 2014;10:32 44. 24149893 10.4161/auto.26508 PMC4389879

[8] Lane CA Hardy J Schott JM Alzheimer's disease. Eur J Neurol 2018;25:59 70. 28872215 10.1111/ene.13439

[9] Larrieu S Letenneur L Orgogozo J Incidence and outcome of mild cognitive impairment in a population-based prospective cohort. Neurology 2002;59:1594 9. 12451203 10.1212/01.wnl.0000034176.07159.f8

[10] Hänninen T Hallikainen M Tuomainen S Prevalence of mild cognitive impairment: a population-based study in elderly subjects. Acta Neurol Scand 2002;106:148 54. 12174174 10.1034/j.1600-0404.2002.01225.x

[11] Briggs AM Cross MJ Hoy DG Musculoskeletal health conditions represent a global threat to healthy aging: a report for the 2015 World Health Organization World Report on Ageing and Health. The Gerontologist 2016;56 Suppl 2:S243 255. 26994264 10.1093/geront/gnw002

[12] Alzheimer's Association. 2016 Alzheimer's disease facts and figures. Alzheimer's Dement 2016;12:459 509. 27570871 10.1016/j.jalz.2016.03.001

[13] Pachon-Angona I Refouvelet B Andrys R Donepezil + chromone + melatonin hybrids as promising agents for Alzheimer's disease therapy. J Enzyme Inhib Med Chem 2019;34:479 89. 30712420 10.1080/14756366.2018.1545766 PMC6366423

[14] Tricco AC Soobiah C Berliner S Efficacy and safety of cognitive enhancers for patients with mild cognitive impairment: a systematic review and meta-analysis. CMAJ 2013;185:1393 401. 24043661 10.1503/cmaj.130451 PMC3826344

[15] Han JY Besser LM Xiong C Cholinesterase inhibitors may not benefit mild cognitive impairment and mild Alzheimer disease dementia. Alzheimer Dis Assoc Disord 2019;33:87 94. 30633043 10.1097/WAD.0000000000000291 PMC6542289

[16] Anand R Gill KD Mahdi AA Therapeutics of Alzheimer's disease: past, present and future. Neuropharmacology 2014;76 (Pt A):27 50. 23891641 10.1016/j.neuropharm.2013.07.004

[17] Li W Kong L-h Wang H High-frequency electroacupuncture evidently reinforces hippocampal synaptic transmission in Alzheimer's disease rats. Neural Regen Res 2016;11:801 6. 27335565 10.4103/1673-5374.182708 PMC4904472

[18] Huang Q Luo D Chen L Effectiveness of acupuncture for Alzheimer's disease: an updated systematic review and meta-analysis. Curr Med Sci 2019;39:500 11. 31209824 10.1007/s11596-019-2065-8

[19] Yu CC Ma CY Wang H Effects of acupuncture on Alzheimer's disease: evidence from neuroimaging studies. Chin J Integr Med 2019;25:631 40. 30155679 10.1007/s11655-018-2993-3

[20] Chang S Guo X Li G Acupuncture promotes expression of Hsp84/86 and delays brain ageing in SAMP8 mice. Acupunct Med 2019;acupmed-2017-011577. 10.1136/acupmed-2017-011577 31412703

[21] Lai H-C Chang Q-Y Hsieh C-L Signal transduction pathways of acupuncture for treating some nervous system diseases. Evid Based Complement Alternat Med 2019;2909632. 31379957 10.1155/2019/2909632 PMC6657648

[22] Yang J-W Wang X-R Ma S-M Acupuncture attenuates cognitive impairment, oxidative stress and NF-(B activation in cerebral multi-infarct rats. Acupunct Med 2019;acupmed-2017-011491. 10.1136/acupmed-2017-011491 31166115

[23] Ding N Jiang J Xu A Manual acupuncture regulates behavior and cerebral blood flow in the SAMP8 mouse model of Alzheimer's disease. Front Neurosci 2019;13:37. 30766475 10.3389/fnins.2019.00037 PMC6365452

[24] Carr MF Karlsson MP Frank LM Transient slow gamma synchrony underlies hippocampal memory replay. Neuron 2012;75:700 13. 22920260 10.1016/j.neuron.2012.06.014 PMC3428599

[25] Oddo S Caccamo A Shepherd JD Triple-transgenic model of Alzheimer's disease with plaques and tangles: intracellular Abeta and synaptic dysfunction. Neuron 2003;39:409 21. 12895417 10.1016/s0896-6273(03)00434-3

[26] Billings L Oddo S Green K Intraneuronal Abeta causes the onset of early Alzheimer's disease-related cognitive deficits in transgenic mice. Neuron 2005;45:675 88. 15748844 10.1016/j.neuron.2005.01.040

